# Length and Amino Acid Sequence of Peptides Substituted for the 5-HT3A Receptor M3M4 Loop May Affect Channel Expression and Desensitization

**DOI:** 10.1371/journal.pone.0035563

**Published:** 2012-04-23

**Authors:** Nicole K. McKinnon, Moez Bali, Myles H. Akabas

**Affiliations:** 1 Department of Physiology and Biophysics, Albert Einstein College of Medicine of Yeshiva University, Bronx, New York, United States of America; 2 Departments of Neuroscience and Medicine, Albert Einstein College of Medicine of Yeshiva University, Bronx, New York, United States of America; Sackler Medical School, Tel Aviv University, Israel

## Abstract

5-HT3A receptors are pentameric neurotransmitter-gated ion channels in the Cys-loop receptor family. Each subunit contains an extracellular domain, four transmembrane segments (M1, M2, M3, M4) and a 115 residue intracellular loop between M3 and M4. In contrast, the M3M4 loop in prokaryotic homologues is <15 residues. To investigate the limits of M3M4 loop length and composition on channel function we replaced the 5-HT3A M3M4 loop with two to seven alanine residues (5-HT3A-A_n = 2–7_). Mutants were expressed in *Xenopus laevis* oocytes and characterized using two electrode voltage clamp recording. All mutants were functional. The 5-HT EC_50_'s were at most 5-fold greater than wild-type (WT). The desensitization rate differed significantly among the mutants. Desensitization rates for 5-HT3A-A_2_, 5-HT3A-A_4_, 5-HT3A-A_6_, and 5-HT3A-A_7_ were similar to WT. In contrast, 5-HT3A-A_3_ and 5-HT3A-A_5_ had desensitization rates at least an order of magnitude faster than WT. The one Ala loop construct, 5-HT3A-A_1_, entered a non-functional state from which it did not recover after the first 5-HT application. These results suggest that the large M3M4 loop of eukaryotic Cys-loop channels is not required for receptor assembly or function. However, loop length and amino acid composition can effect channel expression and desensitization. We infer that the cytoplasmic ends of the M3 and M4 segments may undergo conformational changes during channel gating and desensitization and/or the loop may influence the position and mobility of these segments as they undergo gating-induced conformational changes. Altering structure or conformational mobility of the cytoplasmic ends of M3 and M4 may be the basis by which phosphorylation or protein binding to the cytoplasmic loop alters channel function.

## Introduction

Fast synaptic transmission transduces extracellular chemical signals into electrical information through the actions of ligand-gated ion channels (LGIC)^4^ on a time scale of a few milliseconds. Neurotransmitter binding to the extracellular binding site of LGIC results in a conformation change: opening the channel and enabling the flux of permeant ions [1], [2], [3]. The Cys-loop receptor superfamily, also known as the pentameric LGIC family (pLGIC), constitutes a major class of LGIC. It includes receptors activated by acetylcholine (ACh), 5-hydroxytryptamine (5-HT) commonly known as serotonin, γ-aminobutyric acid (GABA) and glycine [2], [4], [5], [6], [7], [8].

All Cys-loop receptor superfamily channels share a common structure. Each receptor is composed of five homologous subunits arranged around a central ion conducting pore [9]. Each subunit contains three domains, 1) an approximately 200 amino acid amino-terminal, extracellular, ligand-binding domain, 2) a transmembrane (TM), ion channel forming domain and 3) an intracellular domain formed mainly by the loop between the M3 and M4 transmembrane segments. In eukaryotic family members, the extracellular domain contains the eponymous Cys-loop, a sequence of 13 amino acids flanked by two disulfide-linked cysteines. The transmembrane domain contains four α-helical membrane-spanning segments (M1, M2, M3, M4). The M2 segments from each subunit line the channel [9], [10], [11], [12]. Short loops connect the M1 and M2 segments and the M2 and M3 segments. In eukaryotic subunits, transmembrane segments M3 and M4 are connected by a large intracellular loop of 50 to 225 amino acids [3], [9].

Genes that code for proteins homologous to eukaryotic Cys-loop receptors were discovered in prokaryotes [13]. This expanded the pLGIC superfamily from one found only in multicellular animals to a superfamily that includes representatives in single cell prokaryotes as well. Despite low sequence identity, the x-ray crystal structures of the GLIC and ELIC prokaryotic homologues demonstrate that they share several key structural features with the eukaryotic family members. These include the β-strand structure of the extracellular domain and the presence of four α-helical membrane-spanning segments [14], [15], [16]. Despite the similarities, the bacterial proteins lack two prominent features of the metazoan Cys-loop receptors. They lack the Cys residues that form the eponymous extracellular Cys-loop, although other residues in the loop are conserved. Furthermore, the bacterial homologues lack the large intracellular M3M4 loop [13], [14], [15], [16]. Instead, sequence alignment and hydrophobicity analysis predict that the prokaryotic proteins have a short intracellular loop of <15 amino acids, with some homologues having a loop as short as 3 amino acids [13].

The structure of the metazoan large intracellular loops is still poorly understood. The amino acid sequence of the M3M4 loop is the least conserved region across the Cys-loop family. In the 4 Å resolution cryoelectron microscopic structure of the nicotinic acetylcholine receptor, only the C-terminal portion of the loop, which forms an α-helix known as the MA helix, was resolved [9]. The recent x-ray structure of the *C. elegans* glutamate-gated chloride channel, GluCl α, provides no insight into the structure of the M3M4 loop because in the construct that was crystallized the M3M4 loop was replaced by a tripeptide, Ala-Gly-Thr [17]. Of note, the GluCl_cryst_ construct that was crystallized does not open with application of the endogenous ligand, glutamate, alone, similar to the properties observed with expression of only GluCl α [18]. It required application of ivermectin or ivermectin plus glutamate to open the channel.

Many functions have been attributed to the M3M4 loop. Residues in the MA helix are important determinants of single-channel conductance in cation selective Cys-loop channels [19], [20], [21], [22] and have been implicated in channel desensitization [23], [24], and Ca^2+^ binding [25], [26], [27]. However, the size-selectivity filter that determines the diameter of the largest permeant cation in the 5-HT3A receptor is located in the transmembrane channel itself and not in the portals into the cytoplasmic vestibule that are lined by the MA helices [28]. Additionally, the loop has been reported to have roles in receptor assembly, targeting, trafficking [29], [30], [31] and functional interactions with other proteins [32], [33].

Despite the large number of functions attributed to intracellular loop residues, replacement of the entire M3M4 loop in the homomeric 5-HT3A and GABA ρ1 receptors with the heptapeptide predicted to form the M3M4 loop in GLIC, a prokaryotic homologue from *Gloeobacter violaceus*, yielded fully functional channels [34]. In contrast, mutant 5-HT3A and GABA ρ1 receptors with the M3 helix connected directly to the M4 helix resulted in receptors that failed to traffic to the cell surface [34]. Similarly, a glycine receptor construct with the GLIC heptapeptide replacing the M3M4 loop was functional [35] as was a nAChR-α7/GluCl-β chimeric construct with much of the M3M4 loop deleted [36]. We infer that the GLIC heptapeptide is sufficiently long to permit the correct orientation and movement of M3 relative to M4 during gating-induced conformational changes. We sought to determine the minimum loop length that would allow receptor function. To test this, we replaced the M3M4 loop of the 5-HT3A receptor with short chains of small amino acids or repeats of alanines (5-HT3A-A_n = 1–7_). We focused on the 5-HT3A receptor because it forms functional homopentamers, and the effects of mutations within the loop on channel conductance have been well characterized. Surprisingly, we found that all mutants containing 2 to 7 alanines were functional, whereas those containing short alternate peptide sequences varied in their overall expression and functionality.

## Materials and Methods

### Mutagenesis and Xenopus oocyte expression

The mouse 5-HT3A cDNA with an N-terminal V5 epitope tag was cloned into the pXOON vector as previously described [34], and is referred to in this paper as “wild-type” (WT). The starting construct for all of the M3M4 loop replacement constructs used in this work was the 5-HT3A-ΔM3M4 construct formed by splicing the cytoplasmic end of M3 (M3-LVHK/QDLQRP…) to the cytoplasmic end of M4 (…WLRVGY/VLDRLL-M4). The splice was made at the site of the “/” to yield the sequence M3-LVHK/VLDRLL-M4 [34]. This removed 115 residues predicted to form the M3M4 loop. The 5-HT3A-ΔM3M4 construct does not yield functional channels [34].

The construction of the 5-HT3A-glvM3M4 chimera with the 5-HT3A M3M4 loop replaced by the GLIC heptapeptide M3M4 loop (SQPARAA) was described previously [34]. Point mutations, alanine and Gly-Ser-Ala insertions into the 5-HT3A-ΔM3M4 construct were generated with the Quikchange site directed mutagenesis procedure (Stratagene, La Jolla, CA). The entire coding region of all mutant constructs was verified by DNA sequencing (Albert Einstein College of Medicine DNA Sequencing Facility). The pXOON plasmid was linearized using NheI and capped mRNA prepared with T7 RNA polymerase (mMessage mMachine, Ambion, Austin, TX).

Use of *Xenopus laevis* in this study was carried out in strict accordance with the recommendations in the Guide for the Care and Use of Laboratory Animals of the National Institutes of Health. The protocol was approved by the Albert Einstein College of Medicine Animal Care and Use Committee (Protocol Number: 20081201). All surgery was performed under tricaine anesthesia, and all efforts were made to minimize suffering. Oocyctes were harvested from *Xenopus* (Nasco Science, Fort Atkinson, WI) anesthetized with 0.15% tricaine. Oocyctes were defolliculated by incubation in 2 mg ml^−1^ Type 1A collagenase (Sigma-Aldrich, St. Louis, MO) in OR2 (85 mM NaCl, 2 mM KCl, 1 mM MgCl_2_, and 5 mM HEPES; pH adjusted to 7.5 with NaOH) for a 60 or 75 min. Oocytes were washed in OR2 and stored at 16°C in SOS medium: 100 mM NaCl, 2 mM KCl, 1 mM MgCl_2_, 1.8 mM CaCl_2_ 5 mM HEPES, pH 7.5 with 100 IU ml^−1^ penicillin, 100 µg ml^−1^ streptomycin, and 250 ng ml^−1^ amphotericin B (Invitrogen, Carlsbad, CA) and 5% horse serum (Sigma-Aldrich). 24 hrs following isolation, oocyctes were injected with 23 nl (2.3 ng) of mRNA and were kept in SOS media for 2–5 d at 16°C.

### Two-electrode voltage clamp recording

Currents were recorded under two-electrode voltage clamp from oocytes 2–5 d post-injection as described previously [37]. Oocytes were continuously superfused under gravity application at 5 ml min^−1^ with calcium-free frog Ringer's buffer (CFFR, 115 mM NaCl, 2.5 mM KCl, 1.8 mM MgCl_2_, 10 mM HEPES, pH 7.5) to which 5-HT was added as required. The perfusion chamber volume was 200 µl. A 3 M KCl/agar bridge connected the ground electrode to the bath. The holding potential was maintained at −60 mV. Glass microelectrodes filled with 3 M KCl had a resistance of less than 2 MΩ. Salts were purchased from Fisher Scientific (Hampton, NH) or Sigma-Aldrich.

The solution exchange rate was determined by the change in oocyte holding current as the bath solution was switched from a NaCl to a KCl containing solution. The solution exchange rate was best fit by a double exponential function (Table 1). The faster component of the solution exchange rate was slightly faster than the desensitization rate for the fastest desensitizing mutant, 5-HT3A-A_5_ (Table 1).

**Table 1:** Functional characteristics of the alanine insertion mutant currents.

Construct	I_max_ (nA)	EC_50_ (µM)	nH	10–90% Activation rise time (ms)	Desensitization time constant τ (s)	n
wildtype	7300±700	0.6±0.02	2.8±0.1	587±51	77±18	3–6
A_7_	7000±850	1.0±0.02	3.7±0.8	881±110*	62±4	3–4
A_6_	6400±1100	2.2±0.03*	2.5±0.3	1017±23**	77±6	3–4
A_5_	2500±500	2.8±0.03**	1.7±0.5	797±108	6±1**	3–5
A_4_	7100±400	0.8±0.07	2.7±0.4	487±25	56±9	3–4
A_3_	6300±800	2.4±0.05*	2.6±0.4	621±47	8±2**	3–4
A_2_	6900±260	1.3±0.04	2.0±0.3	617±32	43±4	3–4
A_1_	1800±380	n/a	n/a	1055±17**	7±1**	3–4
Solution Exchange –fast component#	n/a	n/a	n/a	n/a	5±1 (88±5%)#	4
Solution Exchange –slow component#	n/a	n/a	n/a	n/a	14±2 (12±5%)#	4

* (p<0.05) and

** (p<0.0002) indicates significant difference from WT by one way ANOVA using Dunnett's multiple comparison *post hoc* test. None of the Hill coefficients were significantly different than WT.

# The solution exchange rate was measured by the change in membrane current of a voltage clamped oocyte in response to a switch from Na^+^ to K^+^ containing buffer solution (see Methods section for details). The current change was best fit by a double exponential function. The percent of each component is given.

Peak current amplitudes were the maximum current in the presence of 5-HT minus the resting current in the absence of 5-HT. 5-HT concentration-response relationships were determined by nonlinear regression fit to the Hill equation with Prism 4 software (GraphPad Software) [12]. Desensitization rates were determined by fitting a single exponential decay function to the current data with Prism 4 software (GraphPad Software). Significant differences compared with wild-type were determined by a one-way ANOVA (*p*<0.05) using Dunnett's multiple comparison *post-hoc* test (*p*<0.05). Mean activation times, defined as the time for current to rise from 10% to 90% of its maximum 10 µM 5-HT value, for wild-type and mutant 5-HT3 receptors were measured in 3 oocytes from 2 different batches. One way ANOVA (P<0.05) with *post hoc* Dunnett's Multiple Comparison test was performed using Prism 5.0 (Graphpad software.)

### Transient transfection

HEK293 cells (CRL-1573, ATCC, Manassas, VA) seeded at low density on poly-lysine-coated, 12 mm, thin glass coverslips were transfected 24 h later using a modified calcium-phosphate precipitation technique with 500 ng DNA per well. After 12 h, cells were rinsed with fresh medium (DMEM supplemented with 10% FBS+100 Units ml^−1^ penicillin+100 µg ml^−1^ streptomycin+4 mM L-glutamine) and incubated at 28°C [34]. Recordings were performed 24–48 h later.

### Patch clamp experiments

For patch clamp experiments the coverslips were mounted on an inverted Zeiss IM microscope equipped with epifluorescent illumination. Cells expressing the GFP reporter were selected for patch clamp recording. Pipettes pulled from borosilicate glass capillaries (GC120TF-10, Harvard Apparatus Inc., Holliston, MA) with a tip resistance of 8–13 MΩ when filled with intracellular solution. The recordings were done using outside-out and cell-attached patch configurations. Data was acquired with an EPC9 amplifier using Pulse software, v8.65 (HEKA Instruments Inc., Bellmore, NY). Data were sampled at 20 kHz and filtered at 10 kHz. All point histograms and fits with a sum of two or three Gaussian functions were performed using the *Levenberg*–*Marquardt* algorithm implemented in Qub software [38]. Recordings were idealized using the Baum–Welch algorithm and mean open times values were obtained by averaging event-duration over all events. Representative recordings used for the figures are presented with additional numerical filtering of 1 kHz.

The current-voltage relationship was determined using the outside-out configuration at voltages ranging from −20 mV to −70 mV in 10 mV increments. Pipette solution contained (in mM): 135 CsCl, 1 MgCl_2_, 2 EGTA, 10 HEPES-Cs pH = 7.3. EGTA was used in the pipette solution to minimize channel block by calcium. The bath solution was composed of (in mM): 140 NaCl, 2.8 KCl, 0.5 CaCl_2_, 30 saccharose, 10 HEPES-Na pH = 7.3. For cell-attached recordings, the pipette solution was composed of (in mM):140 KCl, 5.4 NaCl. 1 EGTA, 10 HEPES-K pH = 7.3. The ionic composition of the bath solution was identical but it was supplemented with 15 mM glucose to prevent cell shrinkage. Under our ionic conditions the membrane patch potential should be close to the real voltage across the patch because the resting potential of the cell should be close to zero, the potassium equilibrium potential in our conditions. Patches were continuously perfused at 0.5 ml/min with extracellular solution delivered via a glass pipette located ∼50 µm away from the tip of the patch pipette. A second glass pipette couple to the first allowed a change in perfusion solution by manually moving the second pipette to a position where it flowed onto the patch pipette. The solution exchange rate in these conditions was 500 ms as estimated by the measured 10% to 90% rise-time of the junction potential due to a change in solution ionic composition. For cell-attached recordings, the pipette tip was filled with the solution above and the pipette was backfilled with the same solution containing 50 µM 5-HT. All point histograms of the illustrative traces were fitted with a sum of two or three gaussians and the mean and standard error was plotted as a function of applied voltage. A linear regression was used to estimate the slope conductance of the channel.

Ondansetron (LKT Laboratories, Inc., St. Paul, MN) was prepared as a 1 mM stock solution in water and diluted to the concentrations indicated in the figure legend. Effects of ondansetron were determined in the outside-out recording configuration at a holding potential of −60 mV.

### Western blotting

Three days post-injection, approximately 70 oocytes for each condition were transferred to a dish containing CFFR buffer and reacted with 0.5 mg ml^−1^ sulfo-NHS-LC biotin for 30 min at room temperature to biotinylate cell surface proteins. The oocytes were rinsed with biotin-free buffer and transferred to Tris-NaCl buffer (100 mM NaCl, 20 mM Tris, pH 7.4) to remove excess biotin. Oocytes were lysed by trituration in 20 µl/oocyte Tris-NaCl buffer containing 1% Triton X-100, 0.5% deoxycholate, 10 mM n-ethyl-maleimide and HALT protease inhibitor cocktail (added according the manufacturer's instructions) (Pierce, Rockford, IL), solubilized by rotating at 4°C for 1 h and centrifuged three times (16,000×g, 20 min, 4°C) to remove yolk. An aliquot from each sample was saved for the total protein fraction, while the remaining sample was incubated with streptavidin beads (Pierce) by rotating for 3 h at 4°C. For the membrane protein fraction, the proteins were eluted from the streptavidin beads by incubation at 37°C for 10 min in 4× SDS-sample buffer. Sample buffer was also added to the total protein fraction aliquots. Proteins were separated by SDS-PAGE on a 4–15% Tris-gradient gel (BioRad, Hercules, CA) and transferred to a PVDF membrane (BioRad). The blot was blocked with 5% powdered skimmed milk, and probed using a mouse monoclonal anti-V5 primary antibody (Invitrogen, 1∶5000 dilution), and a goat anti-mouse horseradish peroxidase-conjugated IgG secondary antibody (Pierce, 1∶5000 dilution). The blot was imaged using SuperSignal West Dura Extended Duration Substrate (ThermoScientific) with a Fluorchem 8000 gel imaging system (Alpha Innotech/Cell Biosciences, Santa Clara, CA).

### Radioactive binding assay

150 nM 5-[1,2-^3^H[N]]-HT (23 Ci mmol^−1^)(PerkinElmer) was added to a solution of non-radiolabeled 10 µM 5-hydroxytryptamine (5-HT) (Sigma-Aldrich) in CFFR. Three to eight oocytes expressing either wildtype 5-HT3A, or a 5-HT3A-M3M4 loop alanine replacement construct were incubated in the radiolabeled 5-HT solution at room temperature for 5 min. Assays were terminated by five washes with ice cold CFFR buffer, followed by solubilization of the oocytes individually with 200 µl 5% SDS. Total wash time was 45 s. Bound radiolabeled 5-HT was determined by liquid scintillation counting (Tri-Carb 2910TR Liquid Scintillation Analyzer, PerkinElmer, Waltham, MA). All data points represent the average of at least fifteen individual oocytes derived from three separate oocyte isolations.

To determine the extent of non-specific binding, all binding experiments were performed in parallel with diethylpyrocarbonate-(DEPC)-treated-water injected oocytes or uninjected oocytes. No significant difference was observed between DEPC-treated-water injected or uninjected oocytes and these data sets were pooled to represent background uptake values.

## Results

### The GLIC M3M4 loop is a pentapeptide

**Figure 1:**
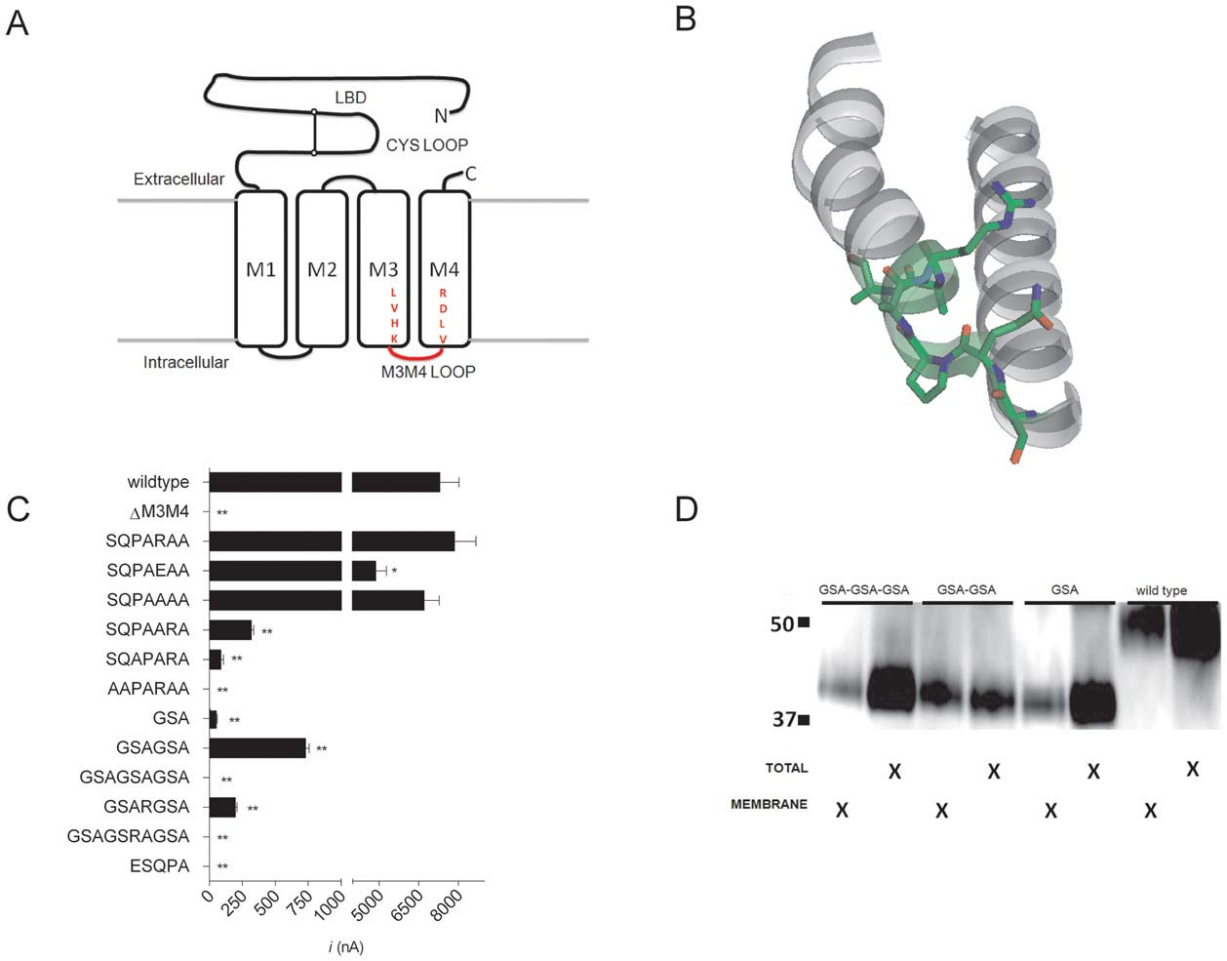
Construct design and initial characterization of 5-HT3A M3M4 loop truncation constructs. (A) Schematic depiction of 5-HT3A truncation constructs. The N-terminal ligand binding domain is connected to the 4 transmembrane segments (open rectangles). M1, M2 and M3 are connected by short loops. The M3M4 cytoplasmic domain including the MA α-helix have been removed and replaced with a short loop (red) corresponding to inserted amino acid linkers. LVHK and VLDR represent the terminal and initial amino acids of the M3 and M4 transmembrane domains, respectively. (B) View of the M3 transmembrane segment thru the M4 segment (including the cytoplasmic M3M4 loop) of a single subunit of the 2.9 Å high resolution crystal structure *Gloeobacter violaceus* GLIC protein (PDB file: 3EAM). The SQPARAA residues that were predicted to form the M3M4 loop are highlighted (green) and the side chains are shown in stick format. Note that the final three residues are part of the M4 α helix on the left. (C) Average peak current recorded from oocyctes expressing 5-HT3A-WT and 5-HT3A-M3M4 loop truncations at a saturating 5-HT (10 µM) concentration. Bars represent the mean ± SEM. The shortened cytoplasmic loops are depicted by the amino acid peptides listed. * (p<0.05) and ** (p<0.0001) indicates peak current significantly different from WT by one way ANOVA using Dunnett's multiple comparison *post hoc* text. (D) SDS-PAGE/Western blot analysis of total and plasma membrane protein fractions from oocyctes expressing(GSA)_n = 1–3_ insertion constructs. 5-HT3A-V5-wild type protein (53 kDa) and 5-HT3A-V5-(GSA)_n = 1–3_ (41 kDa) bands are observed.

Sequence alignment and hydropathy plot analysis of pLGIC/Cys-loop superfamily members predicted that the M3M4 loop of the prokaryotic *Gloeobacter violaceus* ligand gated ion channel homologue, GLIC, is a heptapeptide with sequence SQPARAA [13]. Replacement of the homomeric 5-HT3A and GABA ρ1 M3M4 loop with the predicted GLIC heptapeptide resulted in functional channels, with characteristics similar to WT receptors (Fig. 1A, C) [34].

Subsequent publication of high resolution crystal structures of the GLIC channel indicated that the M3M4 intracellular loop is shorter and starts one residue closer to the N-terminus (ESQPA) than the loop predicted by sequence alignment and hydrophobicity analysis [13], [15], [16] (Fig. 1B). Furthermore, the final three residues in the predicted loop, Arg-Ala-Ala are the initial three residues of the GLIC M4 α-helix in the crystal structure. We investigated whether a 5-HT3A receptor with the crystallographically defined GLIC M3M4 loop pentapeptide (ESQPA) replacing the 5-HT3A M3M4 loop would be functional. Following expression of WT receptors in oocytes, application of 10 µM 5-HT, a saturating concentration, resulted in large inward currents, 7300±700 nA (n = 6). In contrast, no currents were observed in oocytes expressing the ESQPA-M3M4 loop construct in response to up to 50 µM 5-HT (Fig. 1C). Thus, we sought to understand how the length and amino acid composition of a truncated M3M4 loop affected 5-HT3A receptor function.

To investigate the unique features of the SQPARAA heptapeptide that permitted full receptor function we made point mutations in the heptapeptide sequence (Fig. 1C). To investigate whether the arginine played a critical role in functional expression, we reversed the charge from positive to negative (SQPA**E**AA) and also replaced the larger Arg with a smaller, neutral Ala (SQPA**A**AA). Both mutations yielded functional receptors, with large currents elicited upon application of 10 µM 5-HT (Fig. 1C). This indicated that the positively charged residue was not essential for the overall function observed in the 5-HT3A-glvM3M4 heptapeptide receptor. It also indicated that a negatively charged amino acid was tolerated in the loop.

While the Arg was not essential, receptor function was very sensitive to its position in the heptapeptide. Moving the Arg one position towards the C-terminus (SQPA**AR**A) reduced the peak current by more than 20-fold to 320±25 nA (n = 5) (Fig. 1C). Moving both the Arg and the Pro one position toward the C-terminus (SQ**APAR**A) caused an additional 3.5-fold decrease in the peak current to 87±30 nA (n = 5; paired t-test, p<0.05) (Fig. 1C). Furthermore, mutating the initial Ser-Gln to Ala-Ala (**AA**PARAA) resulted in non-functional channels (Fig. 1C). Thus, the relative position of the specific amino acids appeared to be very important in determining whether a specific heptapeptide M3M4 sequence yielded functional receptors. Because the results from this approach seemed difficult to interpret we tried an alternative approach, replacing the heptapeptide with seven alanines.

### Alanine repeats in the M3M4 loop result in functional receptors

**Figure 2:**
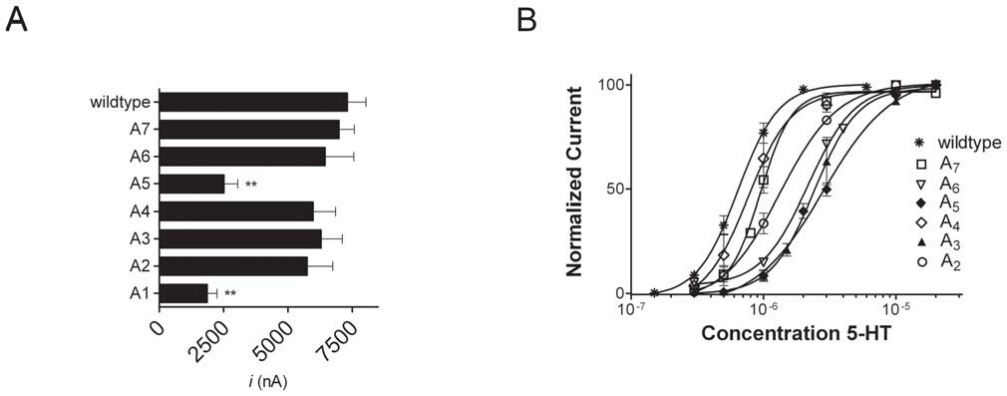
5-HT concentration-response relationships of the 5-HT3A-A_n = 1–7_ constructs are comparable to WT. (A) Peak current recorded from oocytes expressing 5-HT_3A_ wild type and 5-HT3A-A _n = 1–7_ loop constructs at a saturating 5-HT (10 µM) concentration. The shortened cytoplasmic loops are depicted by the number of alanines [A] present between the M3 and M4 transmembrane domains. (B) Concentration-response relationship for 5-HT activation of the 5-HT3A-A_n = 1–7_ constructs (A7 □, A6 ▿, A5 ⧫, A4 ⋄, A3 ▴, A2 ○ and WT <$>\raster="rg1"<$>). Currents were normalized to the maximum response for individual oocytes (n = 3–6). Mean ± SEM shown. ** indicates peak current significantly different from WT by one way ANOVA using Dunnett's multiple comparison *post hoc* test (p<0.0001).

Given the results described above, we were surprised to discover that replacing the WT M3M4 loop with a seven alanine (A_7_) peptide yielded functional channels with currents (7,000±850 nA) comparable to WT (Fig. 2, Table 1). We sought to use this construct to investigate the role of M3M4 loop length on function. We started with the non-functional 5-HT3A-ΔM3M4 construct (Fig. 1C). To determine the minimal number of amino acids required to yield a functional 5-HT3A receptor we generated constructs inserting one to seven alanines (A_n_ where n = 1–7) between the putative cytoplasmic ends of the M3 and M4 transmembrane segments. Following expression in oocytes, application of 10 µM 5-HT induced inward currents for all seven alanine insertion mutants (Fig. 2A and Table 1). The 5-HT EC_50_ and Hill slopes were determined for each of the alanine insertion mutants (Fig. 2B and Table 1), with the exception of the mutant that contained one alanine in the M3M4 loop. For the 5-HT3A-A_1_ construct, as will be described later in detail, the first 5-HT application induced a current response but further applications of 5-HT did not elicit a significant current response in a given oocyte. For WT 5-HT3A receptors the 5-HT EC_50_ was 0.6±0.02 µM. For the alanine insertion mutants the 5-HT EC_50_ ranged from 0.8±0.07 µM (5-HT3A-A_4_) to 2.8±0.03 µM (5-HT3A-A_5_). Thus the minimal linker required in the 5-HT3A M3M4 cytoplasmic loop to obtain functional receptors, with a concentration-response relationship similar to WT, was two alanine residues.

### Alanine repeats 3 and 5 demonstrate markedly faster desensitization kinetics

**Figure 3:**
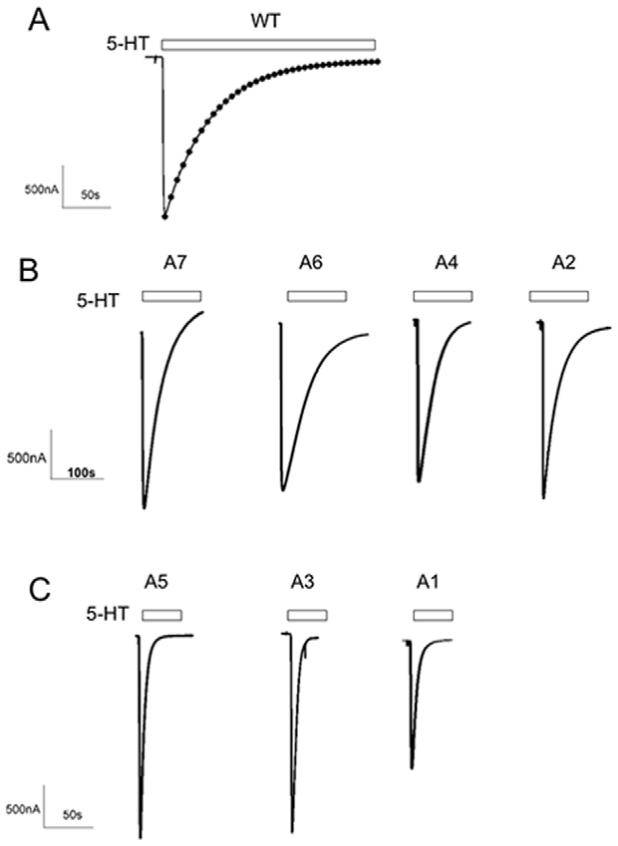
5-HT3A-A_n = 1,3,5_ receptors desensitize more rapidly that 5-HT3A-A_n = 2,4,6,7_. Representative traces for oocytes expressing 5-HT3A- (A) WT (B) A7, A6, A4, A2 (C) and A5, A3, A1 receptors during prolonged exposure to 10 µM 5-HT. (A) The desensitizing component of the current from an oocyte expressing WT 5-HT3A receptors is fit by a single exponential decay function. The dots represent the fitted line which overlays the current trace. (A). Note the difference in the time scale between panels (B) and (C). The A5, A3, and A1 receptors desensitized an order of magnitude faster than A7, A6, A4 and A2 receptors which were similar to WT.

Prolonged exposure to agonist results in desensitization of most Cys-loop receptors. The structure of the desensitized state(s) is not well understood. We observed that some of the alanine insertion constructs desensitized much faster than wild type (Fig. 3). We measured the desensitization rate for each construct simply to demonstrate quantitatively that the observed differences were significant. For all constructs the desensitizing currents in oocytes were well fit by a single exponential decay function (Fig. 3A). For wild type the desensitization time constant was 77±18 s, similar to that reported in the literature for 5-HT3A receptors expressed in oocytes [39], [40]. The desensitization time constants for the A_2_, A_4_, A_6_ and A_7_ constructs were similar to WT (Table 1, Fig. 3B). In contrast, the desensitization time constants for the 5-HT3A-A_1_, 5-HT3A-A_3_, and 5-HT3A-A_5_ alanine insertion constructs were at least ten-fold faster than WT (Table 1, Fig. 3C). Our measured desensitization rates for these constructs may be limited by the solution exchange time in our perfusion system. In terms of our interpretation of these results we wish to focus on the qualitative difference not on the absolute desensitization rates. Our results show that the desensitization time constants for the 5-HT3A-A_1_, 5-HT3A-A_3_, and 5-HT3A-A_5_ constructs were significantly faster than the desensitization time constants for wild type and the other alanine insertion constructs. We do not draw any inferences from the absolute values of the desensitization time constants. For this reason we did not bother to use a faster perfusion system, which we recognize would have allowed measurement of faster components of the desensitization processes [23], [41], [42], [43], [44], [45].

**Figure 4:**
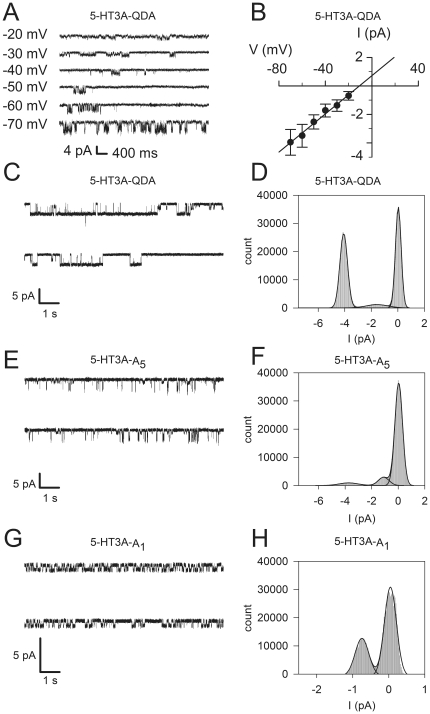
Characterization of 5-HT3A-QDA and 5-HT3A-A_n_ receptors in transfected HEK 293 cells using patch clamp recording. (A) Current/voltage relationship of 5-HT3A-QDA receptors. Representative traces from outside-out patch held at various negative voltages used for the construction of the I/V relationship in panel B. (B) Mean current amplitudes, as determined from all points histograms fitted with a sum of Gaussian functions, are plotted as a function of holding voltage. The line is the linear regression fit to the data points and its slope gives the slope conductance of the channel, 67±7 pS (n = 5). (C, E, G) Single channel recordings from a cell-attached patches containing 5-HT3A-QDA (C) channels, 5-HT3A-A_5_ (E) and 5-HT3A-A_1_ (G) at −60 mV in the presence of 50 µM 5-HT. (C) The mean open time for the main conductance is 103±7 ms (n = 5). Note that occasional subconductance levels are visible but rare. Channel openings are represented as downward deflections. Records filtered at 1 kHz for display purposes. (E) The recordings from 5-HT3A-A_5_ showed a larger conductance with shorter openings, mean open time = 8±2 ms (n = 4), and a smaller sub-conducting state with a mean open time of 48±5 ms. (D, F, H) All-points histograms of the recording on the left were fitted with three Gaussian functions representing the closed and open channel levels and are shown for 5-HT3A-QDA (D) channels, 5-HT3A-A_5_ (F) and 5-HT3A-A_1_ (H).

We used single channel recordings from transiently transfected HEK-293 cells to further characterize a subset of the constructs. For the WT control, we used the 5-HT3A-QDA construct because the single channel conductance of WT 5-HT3A channels is too small to measure [19], [20]. The single channel conductances were determined from the slope of the current-voltage relationship obtained from outside-out patches (Fig. 4B). In the outside-out configuration, the major conductance state for the 5-HT3A-QDA receptor was 67±5 pS (n = 3) (Fig. 4A, B). A subconductance state of 26.3±4.7 pS was also observed (Fig. 4A). Varying single channel conductances have been reported for the 5-HT3A-QDA mutant ranging from about 40 pS [20], [21], [37] to 60 pS [44]. The differences in reported single channel conductance may be due to differences in patch configuration and solution composition [20], [21], [37], [44]. In 4 patches from cells transfected with empty vector, no similar 5-HT activated channels were observed (data not shown). Furthermore, no similar channels were observed when 5-HT was applied in the presence of 1 µM ondansetron (data not shown).

In the mutant A_1_, A_5_ and A_7_ receptors, two conductance levels were also observed. In all three constructs the larger conductance was not significantly different than the WT main state conductance. In contrast, the subconductance levels varied among the constructs; 17.0±0.9 pS in A_1_, 28.5±3.0 pS in A_5_ (Fig. 4C–H) and 9.5±1.0 pS in A_7_ (data not shown). The large conductance was rarely seen in the A_1_ receptor. Previous studies have reported observing subconductance states in recordings from 5-HT3A-QDA receptors [37], [44].

The 5-HT3A-A_5_ receptor displayed extremely short lived opening events in comparison to the 5-HT3A-QDA receptor. The mean channel open time for 5-HT3A-WT-QDA channels was 103±7 ms (n = 5). For the 5-HT3A-A_5_ receptor the mean open time for the larger conductance openings was significantly shorter, 8±2 ms (n = 4), and the small sub-conductance state had a mean open time of 48±5 ms. The short lifetime of the open events could be due to an increase in the closing rate or an increase in the rate of desensitization. Our current single channel experiments cannot distinguish between these two possibilities. However, our macroscopic current recordings suggest that the short open state lifetime may be due to an increase in the desensitization rate.

### After the initial opening, the 5-HT_3A_-A_1_ receptor enters a non-functional, non-binding state

**Figure 5:**
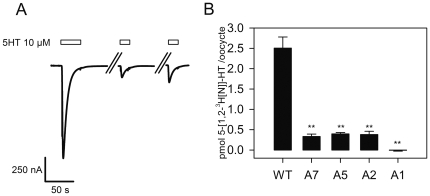
Characterization of the 5-HT3A-A_1_ insertion construct. (A) Currents elicited by repeated applications of 10 µM 5-HT are significantly smaller than the current following the initial 5-HT application in the 5-HT3A-A_1_ receptor. The wash time denoted by the//was 6 min. Insert shows an expanded time scale of the indicated region. The second current response amplitude was 11.6% of the initial response. (B) Radioactive 5-HT binding to oocytes expressing 5-HT3A-WT and 5-HT3A-A_n_ insertion constructs was determined by subtracting 5-HT binding to water-injected oocytes from 5-HT binding to channel expressing oocytes. Each bar represents the mean 5-HT binding ± SEM for at least 10 channel expressing oocytes from at least three separate oocyte isolations. ** indicates radioactive 5-HT binding significantly different from WT by one way ANOVA using Dunnett's multiple comparison *post hoc* test (p<0.0001).

For the 5-HT3A-A_1_ construct expressed in oocytes, the initial 5-HT application elicited a significant current (1800±380 nA; n = 4) that rapidly decayed towards the baseline with a time constant of 7±1 s (Fig. 5A and Table 1). However, subsequent 5-HT applications 360 s later induced very small currents (159±5 nA; n = 4), about 10% of the initial response (Fig. 5A). Increase the wash interval to 10 min did not result in significantly larger second responses. The small subsequent currents might arise from channels recovering from desensitization. However, wild type 5-HT3A channels recover within 30 s at room temperature [45]. This makes it unlikely that this represents recovery from the normal desensitized state. Furthermore, because membrane recycling occurs between the oocyte plasma membrane and internal membrane compartments we cannot rule out the possibility that the small currents observed after prolonged washes (10 to 20 minutes) were due to new channels inserted into the plasma membrane that were not on the membrane surface at the time of the first 5-HT application. We generated constructs where the single alanine was replaced by either a single glycine or proline but neither construct generated any 5-HT induced currents (data not shown).

We hypothesized that given the rapid decay of the initially evoked currents, the truncated 5-HT3A-A_1_ receptors might be entering a non-functional state unrelated to the structural/functional states accessible to a normal full-length 5-HT3A receptor. Alternatively, the 5-HT3A-A_1_ receptors might be entering a desensitized state from which they did not recover on our experimental time scale. If the 5-HT3A-A_1_ receptors were in a desensitized state following the initial 5-HT application they should bind 5-HT with high affinity. If they were in a non-functional state then we would not expect 5-HT to remain bound with high affinity. To test this we measured [^3^H]-5-HT binding to oocytes expressing WT or alanine insertion mutant 5-HT3A receptors. The oocytes were incubated at room temperature with a saturating concentration of [^3^H]5-HT for 5 minutes so that most of the receptors should be in a high affinity desensitized state. The wash steps to remove unbound [^3^H]-5-HT were performed at 4°C to slow recovery from desensitization and completed in 45 s. Although the Q_10_ for recovery from desensitization has not been measured in 5-HT3A receptors, in the homologous nACh receptors it is about 2 [46]. Thus, with a time to recovery from desensitization at room temperature of 30 s in 5-HT3A receptors [45], the time to complete recovery at 4°C should be about 120 s, significantly longer than the 45 s wash period in our experiments. Thus, many of the receptors should remain in a desensitized state throughout the wash period.

Using this protocol, oocytes expressing WT receptors bound 2.50±0.3 pmol of [1,2-^3^H[N]]5-HT per oocyte. The 5-HT3A-A_7_, 5-HT3A-A_5_, 5-HT3A-A_2_ constructs bound 0.40±0.08, 0.40±0.03 and 0.33±0.06 pmol of 5 [1,2-^3^H[N]]-HT per oocyte, respectively. This difference in binding between the 5-HT3A-A_7_, 5-HT3A-A_5_, 5-HT3A-A_2_ constructs, and WT receptors is likely due to different expression levels of these constructs in the oocyte plasma membrane. Surprisingly, the 5-HT binding to 5-HT3A-A_1_ receptor expressing oocytes was undetectable, with radioactivity counts identical to uninjected or DEPC-water injected oocytes (Fig. 5B). The lack of detectable 5-HT bound to the 5-HT3A-A_1_ construct was statistically significantly different (p<0.05) from all of the other receptor constructs tested. We do not think that the lack of detectable 5-HT binding to oocytes expressing the 5-HT3A-A_1_ construct was due to markedly reduced levels of 5-HT3A-A_1_ receptor expression because the initial 5-HT induced currents for the 5-HT3A-A_1_ expressing oocytes were about 70% of the level of the 5-HT3A-A_5_ construct expressing oocytes (Table 1) for which there was detectable binding. Thus, we conclude that after exposure to 5-HT the 5-HT3A-A_1_ construct is not in a high affinity state. This allows the [^3^H]5-HT to dissociate from the receptor during the wash period. We infer that after the initial 5-HT application the 5-HT3A-A_1_ channel enters a low affinity, non-functional state from which it cannot reopen on a meaningful time scale. We believe that this state is not relevant to the structural/functional states that are accessible to normal, full-length 5-HT3A receptors.

### The M3M4 loop sequence can determine receptor functionality

In some of our preliminary experiments we replaced the M3–M4 loop in the 5-HT3A-ΔM3M4 construct with repeats of the tripeptide Gly-Ser-Ala (GSA). We chose this sequence because the amino acid side chains were small and we thought it would provide the backbone flexibility necessary to make the turn between the α helical M3 and M4 transmembrane segments. Curiously, while the replacement of the M3M4 loop with two to seven alanines was well tolerated, insertion of repeats of the GSA tripeptide were poorly tolerated. As with the alanine insertions, we started with the non-functional 5-HT3A-ΔM3M4 construct. We inserted repeats of the small, amino acids (GSA)_n = 1–3_ into the fusion site. Two electrode voltage clamp recording from oocytes expressing the GSA M3M4 mutants revealed low 5-HT induced currents for the GSA (50±12 nA) and GSAGSA (730±45 nA) mutants (n = 3) (Fig. 1C). In contrast, there were no detectable currents from oocytes (n = 3) expressing the GSAGSAGSA mutant (Fig. 1C). Given the role of arginines in the cytoplasmic loops for transmembrane topology [47], we created two additional GSA_n = 2–3_ insertion mutants, each with an arginine present in the M3M4 loop linker. The addition of the arginine did not restore a WT level of 5-HT induced current to either mutant (Fig. 1C).

We assayed protein expression in whole oocyte and plasma membrane fractions using Western blots probed with an anti-V5 primary antibody. Membrane protein fractions were isolated by surface biotinylation and subsequent pull down with streptavidin-agarose beads. For each GSA_n = 1–3_ insertion construct and for the WT receptor, bands corresponding with the expected protein sizes were detected in both the total protein and plasma membrane fractions (Fig. 1D). The amount of receptor protein was similar in WT and in the (GSA)_1_ and (GSA)_3_ constructs. However, the level of plasma membrane expression was reduced for the mutants compared to WT and was roughly in proportion to the relative current amplitudes (GSA)_2_>(GSA)_1_>(GSA)_3_ (Fig. 1D). This suggested that the M3M4-loop GSA-insertions were synthesized at comparable levels to WT but the trafficking to the surface membrane may have been impaired. This implies that receptor function and plasma membrane trafficking is sensitive to the amino acid composition of the inserted residues. Thus, the choice of residues substituted into the M3–M4 loop can affect trafficking to the plasma membrane. We do not understand the mechanistic basis for this observation nor the amino acid sequence dependence for this phenomenon.

## Discussion

In Cys-loop receptor family members, the cytoplasmic M3M4 loop is the most variable portion of the proteins. In eukaryotic subunits, it is greater than 75 residues in length but in the prokaryotic subunits, it is less than 15 residues in length. We previously showed that chimeras, where the 5-HT3A or GABA ρ1 M3M4 loop was replaced by the predicted loop sequence of the prokaryotic GLIC subunit, were functional [34]. Other investigators have subsequently shown that substitution of the GLIC heptapeptide in the homopentameric glycine receptor also results in functional channels [35]. In the present work we started with a non-functional 5-HT3A-ΔM3M4 construct from which the 115 residue M3M4 loop was excised and the C-terminus of M3 was coupled to the N-terminus of M4. We explored the effect on receptor function of inserting various peptides into the junction between the M3 and M4 segments to determine the minimal loop necessary for functional receptors. This provides information on the distance separating the cytoplasmic ends of the M3 and M4 segments and the extent to which they move during channel gating.

To explore the effect of loop length, we inserted between one and seven alanines between the M3 and M4 segments in the 5-HT3A-ΔM3M4 construct. All of the constructs with between two and seven alanines yielded functional 5-HT responsive channels when expressed in oocytes. The most striking functional difference between the constructs was the effect of loop length on the desensitization rate. The constructs with three or five alanines desensitized an order of magnitude faster than wild type or the two, four, six or seven alanine constructs. The alternating pattern suggests that the inserted alanines may adopt a β strand secondary structure where the odd numbers of inserted alanines creates strain on the position of the cytoplasmic ends of the M3 and M4 segments or limits their ability to move during channel gating resulting in the altered desensitization rates. Beyond six alanines, the loop length may be sufficiently long that the β strand secondary structure either does not occur or is no longer a constraint on movement of M3 or M4 during desensitization. However, it should be noted that loops longer than seven residues are not necessarily sufficient to ensure normal function, it may also depend on the amino acid composition of the loop. The fact that the loop length affects the desensitization rate implies that the cytoplasmic ends of the M3 and M4 membrane-spanning segments must undergo a conformational change during desensitization. This may induce conformational changes in the endogenous loop during desensitization. Alternatively, the loop may constrain the position of the M3 and M4 segments in the overall protein structure in such a way that it results in more rapid desensitization of the resultant channels. Work by Vogel and colleagues showed agonist-induced changes in homo-FRET between EGFP molecules inserted into the 5-HT3A receptor M3M4 loop [48]. They inferred that desensitization induces a conformational change in the M3M4 loop structure [48]. Our results support the inference from the homo-FRET experiments that the cytoplasmic ends of the M3 and M4 segments move during desensitization and that, at least desensitization, induces a conformational change in the 5-HT3A M3M4 loop.

Other investigators have shown that mutations of residues in the endogenous M3M4 loop can also alter the desensitization rates [23]. In addition, the binding of proteins to the 5-HT3 M3M4 loop or phosphorylation of residues within the loop may lead to modulation of desensitization rates [49], [50]. Both extracellular and intracellular calcium concentrations have profound effects on 5-HT3A receptor desensitization rate [39], [42], [51]. Our experiments were performed in nominally Ca^2+^-free buffer. This accounts, at least in part, for the significantly slower desensitization rates that we observed (Table 1) than those reported in the literature from recent patch clamp studies of channel kinetics [44], [45]. 5-HT3 desensitization rate may have significant impact on normal physiological functions in the brain. A 5-HT3B polymorphism, Y129S, causes a 10-fold slowing in desensitization rates. This polymorphism has been associated with a reduced incidence of major depression in woman [52]. Thus, modulation of desensitization rates may serve as a means of *in vivo* regulation of 5-HT3 receptor function.

The amino acid sequence of the loop also impacts function. Thus, insertion of nine residues, three repeats of Gly-Ser-Ala, yielded non-functional receptors and insertion of six residues, GSAGSA, yielded poorly functioning receptors. Thus, glycines that would be expected to confer increased flexibility in the loop were not as well tolerated as alanines. At present, we do not understand how the insertion of Gly and Ser residues into the loop has such a significant effect on receptor expression/function but this study suggests that if one wishes to remove the native M3M4 loop to generate potential constructs for crystallization trials then the nature of the residues substituted for the M3M4 loop may have a large impact on expression/function.

**Figure 6:**
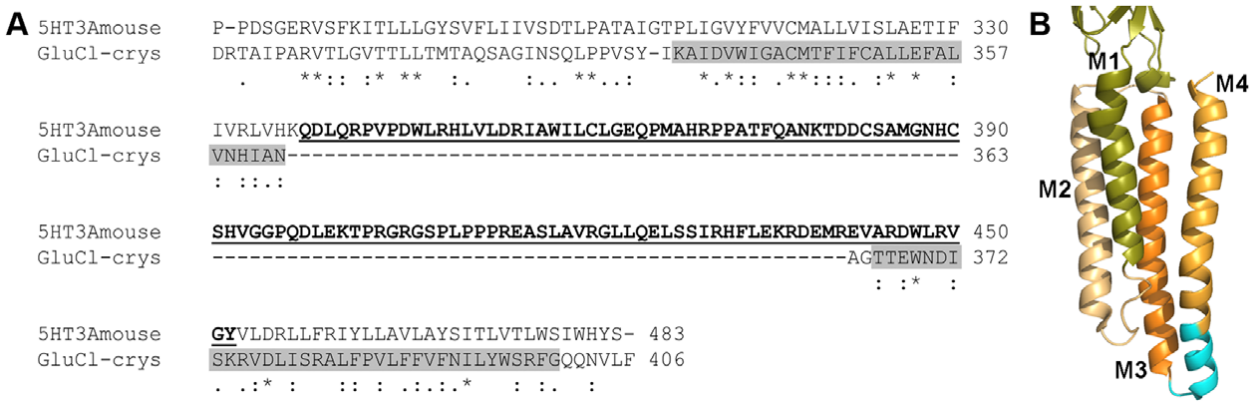
Sequence alignment GluCl_crys_ and 5-HT3A and cartoon illustrating transmembrane region of GluCl_crys_. (A) Sequence alignment of the C-terminal region of mouse 5-HT3A receptor and GluCl_crys_. The GluCl_crys_ M3 and M4 α helical membrane-spanning segments are indicated by the gray rectangles. The M3M4 loop region replaced by two alanines in the 5-HT3A-A_2_ construct is shown underlined in bold. The 5-HT3A-A_2_ construct yielded functional receptors with channel kinetics similar to wild type 5-HT3A receptors. Note that the 5-HT3A-A_2_ construct lacks 16 residues aligned with the cytoplasmic end of the GluCl M4 segment. (B) Cartoon representation of the transmembrane domain of one subunit of GluCl (PDB: 3RHW). The cyan region at the cytoplasmic end of the M4 segment indicates the residues that were replaced by two alanine residues in the5-HT3A-A_2_ construct.

It is instructive to compare our 5-HT3A constructs lacking the M3M4 loop with the GluCl_crys_ construct used in the recent crystallization study [17]. The cytoplasmic ends of the M3 segments differ by one residue between our 5-HT3A construct and GluCl_crys_ (Fig. 6A). In contrast, the junction site at the cytoplasmic end of the M4 segment differs by 16 residues (Fig. 6A, B). Our 5-HT3A-A_2_ construct that displayed near wild type expression and functional properties lacks the residues forming the cytoplasmic end of the M4 segment in the CluCl_crys_ structure (Fig. 6B, cyan region of M4). Similarly, an M3M4 loop deletion construct in a nAChR-α7/GluCl-β chimeric construct displayed similar function to the full-length construct despite lacking some of the residues that form the cytoplasmic end of the GluCl_crys_ construct M4 segment [36]. This suggests that the structure in this region may be quite flexible. Perhaps the structure of this region seen in the GluCl crystal is determined in part by the splice sites chosen in developing the construct. Alternatively, though less likely, the structure of the cytoplasmic ends of M3 and M4 are quite different in the 5-HT3A receptor than in GluCl_crys_. Further experiments will be necessary to understand how the presence of the M3M4 loop or various deletions affect the protein structure in this region.

The ability to replace the 115 residue M3M4 loop with as few as two alanines and still obtain high level expression of functional 5-HT_3A_ receptors implies that the endogenous loop is not essential for homopentamer assembly in the endoplasmic reticulum (ER). While ER chaperone proteins may be involved in 5-HT3A receptor folding and assembly [53], our results imply that interactions with cytoplasmic chaperone proteins are not essential for the folding and assembly process. Furthermore, the ability of the alanine loop constructs to traffic from the ER to the plasma membrane suggests that this process, at least in *Xenopus* oocytes, also does not require interactions with cytoplasmic proteins. This suggests that export of alanine loop receptors to the plasma membrane either only requires interactions with the extracellular and transmembrane domains or occurs via a default trafficking pathway.

The initial current elicited by application of 5-HT to the construct containing a single alanine decayed rapidly compared to wild type. However, this receptor never recovered to a conformation from which it could be re-opened on the time scale of our experiments. The results from the [^3^H]-5-HT binding experiments show that the receptor does not remain in a high affinity, ligand-bound, desensitized state. Furthermore, because the receptor is unable to enter the open conformation with subsequent 5-HT applications, we suggest that the receptor is not in the resting-activatable closed conformation either. We propose that the receptor is in a non-ligand bound, non-functional state. We previously suggested that a possible explanation for why the 5-HT3A-ΔM3M4 construct was non-functional was that the M4 helix might be unable to insert into the membrane during protein synthesis [34]. The single alanine insertion along with one or two residues from the ends of M3 and M4 may be flexible enough to permit the M4-helix to enter the membrane, but not flexible enough to permit the conformational changes that must normally occur in this region during the transition from the open to the desensitized state. Thus, with a single alanine the receptor can open to a metastable state that decays into a non-functional state that is not relevant to the normal close, open and desensitized states that the WT receptor transitions through.

In the present study we have defined the minimum length of the M3M4 loop necessary to obtain functional 5-HT3 receptors and the effects of amino acid composition on expression and function. The fact that all eukaryotic Cys-loop receptor subunits have M3M4 loops >75 residues in length, far longer than the minimum required, suggests that the longer loops have evolved to fulfill important functions for these neurotransmitter-gated channels. Elucidating the structure of these loops will provide new insight into their functional roles and their interactions with cytoplasmic proteins involved in the regulation and localization of these channels.

## References

[1] Akabas MH (2004). GABA_A_ receptor structure-function studies: a reexamination in light of new acetylcholine receptor structures.. Int Rev Neurobiol.

[2] Lester HA, Dibas MI, Dahan DS, Leite JF, Dougherty DA (2004). Cys-loop receptors: new twists and turns.. Trends in neurosciences.

[3] Miller PS, Smart TG (2010). Binding, activation and modulation of Cys-loop receptors.. Trends Pharmacol Sci.

[4] Peters JA, Kelley SP, Dunlop JI, Kirkness EF, Hales TG (2004). The 5-hydroxytryptamine type 3 (5-HT3) receptor reveals a novel determinant of single-channel conductance.. Biochem Soc Trans.

[5] Lynch JW (2004). Molecular structure and function of the glycine receptor chloride channel.. Physiol Rev.

[6] Barnes NM, Hales TG, Lummis SC, Peters JA (2009). The 5-HT3 receptor–the relationship between structure and function.. Neuropharmacology.

[7] Macdonald RL, Olsen RW (1994). GABAA receptor channels.. Annu Rev Neurosci.

[8] Thompson AJ, Lester HA, Lummis SC (2010). The structural basis of function in Cys-loop receptors.. Q Rev Biophys.

[9] Unwin N (2005). Refined structure of the nicotinic acetylcholine receptor at 4A resolution.. J Mol Biol.

[10] Imoto K, Busch C, Sakmann B, Mishina M, Konno T (1988). Rings of negatively charged amino acids determine the acetylcholine receptor channel conductance.. Nature.

[11] Xu M, Akabas MH (1996). Identification of channel-lining residues in the M2 membrane-spanning segment of the GABA(A) receptor alpha1 subunit.. J Gen Physiol.

[12] Reeves DC, Goren EN, Akabas MH, Lummis SC (2001). Structural and electrostatic properties of the 5-HT3 receptor pore revealed by substituted cysteine accessibility mutagenesis.. J Biol Chem.

[13] Tasneem A, Iyer LM, Jakobsson E, Aravind L (2004). Identification of the prokaryotic ligand-gated ion channels and their implications for the mechanisms and origins of animal Cys-loop ion channels.. Genome Biol.

[14] Hilf RJ, Dutzler R (2008). X-ray structure of a prokaryotic pentameric ligand-gated ion channel.. Nature.

[15] Bocquet N, Nury H, Baaden M, Le Poupon C, Changeux JP (2009). X-ray structure of a pentameric ligand-gated ion channel in an apparently open conformation.. Nature.

[16] Hilf RJ, Dutzler R (2009). Structure of a potentially open state of a proton-activated pentameric ligand-gated ion channel.. Nature.

[17] Hibbs RE, Gouaux E (2011). Principles of activation and permeation in an anion-selective Cys-loop receptor.. Nature.

[18] Cully DF, Vassilatis DK, Liu KK, Paress PS, Van der Ploeg LH (1994). Cloning of an avermectin-sensitive glutamate-gated chloride channel from Caenorhabditis elegans.. Nature.

[19] Davies PA, Pistis M, Hanna MC, Peters JA, Lambert JJ (1999). The 5-HT3B subunit is a major determinant of serotonin-receptor function.. Nature.

[20] Kelley SP, Dunlop JI, Kirkness EF, Lambert JJ, Peters JA (2003). A cytoplasmic region determines single-channel conductance in 5-HT3 receptors.. Nature.

[21] Hales TG, Dunlop JI, Deeb TZ, Carland JE, Kelley SP (2006). Common determinants of single channel conductance within the large cytoplasmic loop of 5-hydroxytryptamine type 3 and alpha4beta2 nicotinic acetylcholine receptors.. J Biol Chem.

[22] Deeb TZ, Carland JE, Cooper MA, Livesey MR, Lambert JJ (2007). Dynamic modification of a mutant cytoplasmic cysteine residue modulates the conductance of the human 5-HT3A receptor.. J Biol Chem.

[23] Hu XQ, Sun H, Peoples RW, Hong R, Zhang L (2006). An interaction involving an arginine residue in the cytoplasmic domain of the 5-HT3A receptor contributes to receptor desensitization mechanism.. J Biol Chem.

[24] O'Toole KK, Jenkins A (2011). Discrete M3–M4 Intracellular Loop Subdomains Control Specific Aspects of gamma-Aminobutyric Acid Type A Receptor Function.. The Journal of biological chemistry.

[25] Noam Y, Wadman WJ, van Hooft JA (2008). On the voltage-dependent Ca2+ block of serotonin 5-HT3 receptors: a critical role of intracellular phosphates.. J Physiol.

[26] van Hooft JA, Wadman WJ (2003). Ca2+ ions block and permeate serotonin 5-HT3 receptor channels in rat hippocampal interneurons.. J Neurophysiol.

[27] Livesey MR, Cooper MA, Deeb TZ, Carland JE, Kozuska J (2008). Structural determinants of Ca2+ permeability and conduction in the human 5-hydroxytryptamine type 3A receptor.. J Biol Chem.

[28] McKinnon NK, Reeves DC, Akabas MH (2011). 5-HT3 receptor ion size selectivity is a property of the transmembrane channel, not the cytoplasmic vestibule portals.. J Gen Physiol.

[29] Connolly CN (2008). Trafficking of 5-HT(3) and GABA(A) receptors.. Mol Membr Biol.

[30] Kracun S, Harkness PC, Gibb AJ, Millar NS (2008). Influence of the M3–M4 intracellular domain upon nicotinic acetylcholine receptor assembly, targeting and function.. Br J Pharmacol.

[31] Lo WY, Botzolakis EJ, Tang X, Macdonald RL (2008). A conserved Cys-loop receptor aspartate residue in the M3–M4 cytoplasmic loop is required for GABAA receptor assembly.. J Biol Chem.

[32] Lansdell SJ, Gee VJ, Harkness PC, Doward AI, Baker ER (2005). RIC-3 enhances functional expression of multiple nicotinic acetylcholine receptor subtypes in mammalian cells.. Mol Pharmacol.

[33] Millar NS (2008). RIC-3: a nicotinic acetylcholine receptor chaperone.. Br J Pharmacol.

[34] Jansen M, Bali M, Akabas MH (2008). Modular design of Cys-loop ligand-gated ion channels: functional 5-HT3 and GABA rho1 receptors lacking the large cytoplasmic M3M4 loop.. J Gen Physiol.

[35] Moroni M, Biro I, Giugliano M, Vijayan R, Biggin PC (2011). Chloride ions in the pore of glycine and GABA channels shape the time course and voltage dependence of agonist currents.. J Neurosci.

[36] Bar-Lev DD, Degani-Katzav N, Perelman A, Paas Y (2011). Molecular dissection of Cl–selective Cys-loop receptor points to components that are dispensable or essential for channel activity.. J Biol Chem.

[37] Reeves DC, Jansen M, Bali M, Lemster T, Akabas MH (2005). A role for the beta 1-beta 2 loop in the gating of 5-HT3 receptors.. J Neurosci.

[38] Qin F, Auerbach A, Sachs F (1996). Estimating single-channel kinetic parameters from idealized patch-clamp data containing missed events.. Biophys J.

[39] Lobitz N, Gisselmann G, Hatt H, Wetzel CH (2001). A single amino-acid in the TM1 domain is an important determinant of the desensitization kinetics of recombinant human and guinea pig alpha-homomeric 5-hydroxytryptamine type 3 receptors.. Mol Pharmacol.

[40] Yakel JL, Lagrutta A, Adelman JP, North RA (1993). Single amino acid substitution affects desensitization of the 5-hydroxytryptamine type 3 receptor expressed in Xenopus oocytes.. Proc Natl Acad Sci U S A.

[41] Gunthorpe MJ, Peters JA, Gill CH, Lambert JJ, Lummis SC (2000). The 4'lysine in the putative channel lining domain affects desensitization but not the single-channel conductance of recombinant homomeric 5-HT3A receptors.. J Physiol 522 Pt.

[42] Hu XQ, Lovinger DM (2005). Role of aspartate 298 in mouse 5-HT3A receptor gating and modulation by extracellular Ca2+.. J Physiol.

[43] Hu XQ, Lovinger DM (2008). The L293 residue in transmembrane domain 2 of the 5-HT3A receptor is a molecular determinant of allosteric modulation by 5-hydroxyindole.. Neuropharmacology.

[44] Corradi J, Gumilar F, Bouzat C (2009). Single-channel kinetic analysis for activation and desensitization of homomeric 5-HT(3)A receptors.. Biophys J.

[45] Solt K, Ruesch D, Forman SA, Davies PA, Raines DE (2007). Differential effects of serotonin and dopamine on human 5-HT3A receptor kinetics: interpretation within an allosteric kinetic model.. J Neurosci.

[46] Dilger JP, Brett RS, Poppers DM, Liu Y (1991). The temperature dependence of some kinetic and conductance properties of acetylcholine receptor channels.. Biochim Biophys Acta.

[47] Wallin E, von Heijne G (1998). Genome-wide analysis of integral membrane proteins from eubacterial, archaean, and eukaryotic organisms.. Protein Sci.

[48] Ilegems E, Pick H, Deluz C, Kellenberger S, Vogel H (2005). Ligand binding transmits conformational changes across the membrane-spanning region to the intracellular side of the 5-HT3 serotonin receptor.. Chembiochem.

[49] Jones S, Yakel JL (2003). Casein kinase ii (protein kinase ck2) regulates serotonin 5-ht(3) receptor channel function in ng108-15 cells.. Neuroscience.

[50] Sun H, Hu XQ, Emerit MB, Schoenebeck JC, Kimmel CE (2008). Modulation of 5-HT3 receptor desensitization by the light chain of microtubule-associated protein 1B expressed in HEK 293 cells.. J Physiol.

[51] Jones S, Yakel JL (1998). Ca2+ influx through voltage-gated Ca2+ channels regulates 5-HT3 receptor channel desensitization in rat glioma x mouse neuroblastoma hybrid NG108-15 cells.. J Physiol.

[52] Krzywkowski K, Davies PA, Feinberg-Zadek PL, Brauner-Osborne H, Jensen AA (2008). High-frequency HTR3B variant associated with major depression dramatically augments the signaling of the human 5-HT3AB receptor.. Proc Natl Acad Sci U S A.

[53] Boyd GW, Low P, Dunlop JI, Robertson LA, Vardy A (2002). Assembly and cell surface expression of homomeric and heteromeric 5-HT3 receptors: the role of oligomerization and chaperone proteins.. Mol Cell Neurosci.

